# DAYTIME SLEEPINESS AND WEIGHT CHANGE AMONG ADULTS: FINDINGS FROM THE WISCONSIN SLEEP COHORT STUDY

**DOI:** 10.1093/geroni/igac059.1439

**Published:** 2022-12-20

**Authors:** Yin Liu, Jodi Barnet, Erika Hagen, Paul Peppard, Eric Reither, Emmanuel Mignot, David Plante

**Affiliations:** Utah State University, Logan, Utah, United States; University of Wisconsin Madison, Madison, Wisconsin, United States; University of Wisconsin Madison, Madison, Wisconsin, United States; University of Wisconsin Madison, Madison, Wisconsin, United States; Utah State University, Logan, Utah, United States; Stanford University, Palo Alto, California, United States; University of Wisconsin-Madison, Madison, Wisconsin, United States

## Abstract

BMI trajectories are associated with nighttime sleep, but it is less clear how they relate to daytime sleepiness. We examined the association between levels and changes in daytime sleepiness and BMI among men and women using growth curve models among 1047 participants in the Wisconsin Sleep Cohort Study (mean [sd] age = 51.1 [8.0] years at baseline). The outcome variable was BMI (kg/m2). Key predictors were self-reported sleepiness measured by the Epworth Sleepiness Scale (ESS), and the objective Multiple Sleep Latency Test (MSLT) scores at each data collection wave. Men, but not women, who were sleepier had higher BMI levels. Age moderated the association between changes in ESS and MSLT sleepiness and BMI trajectories. The association was weaker for older men, but stronger for younger men; such effect was the opposite for women. The MSLT models further suggested that women who were sleepier had steeper increases in BMI over time.

